# A projection-based approach for clarifying interaction partners in human-robot communication

**DOI:** 10.3389/frobt.2025.1534060

**Published:** 2025-03-27

**Authors:** Suguru Sone, Tsubasa Kishi, Tetsushi Ikeda

**Affiliations:** Graduate School of Information Sciences, Hiroshima City University, Hiroshima, Japan

**Keywords:** guide robots, human-robot interaction, projection-based communication, service robots, social robotics

## Abstract

Robots provide a variety of services in daily life spaces, making human-robot interaction essential. This research proposes a new projection-based method for non-humanoid robots to engage with people. While significant research has explored the use of human-like gestures in humanoid robots to initiate interaction, applying such approaches to non-humanoid robots is challenging in conveying the sense that the robot is addressing the person directly. In this study, we introduce a method where a projector mounted on the robot illuminates the area around both the robot and the partners it is addressing, enhancing the interaction clarity and participation. Experiments conducted in two scenarios demonstrated that the proposed method effectively conveyed the feeling of being directly addressed by the robot and fostered a sense of easy participation, even for those not actively participating.

## 1 Introduction

Robots are expected to coexist with humans and provide services that support human daily life. Research has advanced in various applications, such as exhibit guidance in museums (Burgard et al., 1998; Shiomi et al., 2006; Iio et al., 2019; Rosa et al., 2023), delivering packages (D. Lee et al., 2021), and information provision in settings like shopping malls (Kanda et al., 2009) and airports (Triebel et al., 2016). The ability to address target partners and initiate interaction is essential for robots operating in human-shared environments, and it remains a prominent area of study (Avelino et al., 2021).

Initiating conversations with specific partners in the presence of multiple people is a challenging task for robots. In human communication, we rely on cues such as standing position, body posture, pointing gestures, facial expressions, and eye contact to indicate to others that we are addressing them directly. Many studies have proposed methods for controlling humanoid robots to replicate such human behaviors (Saad et al., 2019; Iio et al., 2019). The importance of designing a coordinated combination of multiple modalities, such as gaze and body movement, has been highlighted (Vázquez et al., 2017; Arai et al., 2019). However, applying these human-like methods to non-humanoid robots presents challenges. To address this issue, studies have examined methods for non-humanoid robots to convey their focus, such as using body orientation (Satake et al., 2013) and gaze direction on displays (Karreman et al., 2013). Nonetheless, accurately conveying the sense that the robot is directly addressing a specific partner or group remains difficult, especially when multiple people are present.

This research proposes a new method in which a non-humanoid robot, which lacks the ability to use gaze or gestures like human- or animal-like robots, uses projection to clearly convey that it is directly addressing multiple parties. Specifically, the robot projects a light field onto the ground that encompasses both the robot and the intended partners, making it explicit whom the robot is addressing. This method is advantageous in providing unambiguous communication, even when the robot is interacting with multiple people, and it is applicable to robots without human-like bodies. This paper builds upon the method proposed by Sone et al. (2022) and validates it through two new experiments conducted on a new projection robot.

The contributions of this research are as follows:

1. We propose a method for a robot to clearly convey its intended interaction partners in environments with multiple people, using an onboard projector to visually highlight them. The proposed method assumes that the robot already knows the positions of the partners it intends to address.
2. We validate the proposed method through two scenarios: a guidance task where the robot sequentially addresses multiple individuals, and an interaction experiment involving multiple partners.
3. In both experiments, subjective evaluations indicated that participants felt the robot was directly addressing them compared to the baseline method, and in Experiment 2, participants reported greater ease in engaging in conversation, with both effects being statistically significant (p < 0.05).

The remainder of this paper is organized as follows: Section 2 reviews related work, Section 3 describes the proposed method, Sections 4 and 5 describe the two validation experiments, and Section 6 presents a discussion of the results. Finally, Section 7 concludes the paper.

## 2 Related work

### 2.1 Research on clarifying whom robots are addressing

Numerous methods have been investigated to enable robots to directly address their intended partners and initiate conversations. Behavioral theories regarding interpersonal distance and spatial positioning in human-human interaction have been proposed and later extended to human-robot interaction. Hall (1966) categorized interpersonal distances in human-human interactions and introduced the concept of proxemics. Kendon (1990) expanded on this by considering not only distance but also spatial formations, proposing that people in public conversations adjust their positions to form specific spatial arrangements. These insights into human conversational dynamics have been leveraged in designing robots that engage effectively in human-robot interactions (Yamaoka et al., 2010). One approach, proposed and experimentally validated by Satake et al. (2013), involves a robot initiating interaction by first approaching a person at an appropriate social distance. They identified that a common cause of unsuccessful interactions is the person’s failure to notice the robot’s intention to start a conversation. To address this, the authors emphasized the need for the robot to clearly and unambiguously signal its intent. Kato et al. (2015) observed natural human behavior in approaching others, focusing on the use of body orientation and gaze, and implemented these behaviors in a mobile robot to evaluate their effectiveness. Similarly, Yang et al. (2020) confirmed that mimicking human approach behaviors is an effective strategy for managing a robot’s movements when approaching a group of people.

When initiating conversation, the importance of using multiple modalities to communicate to people that the robot is attempting to address them directly has been widely recognized. For instance, Saad et al. (2019) demonstrated that when a robot greets a partner entering through a doorway with gestures or vocal cues to attract attention, the number of people who respond to the robot increases, while the number of unresponsive partners decreases. Strait et al. (2014) examined the effects of different modalities when a robot speaks to a person to provide advice, while Hoque et al. (2012) developed a method for recognizing a person’s facial orientation and gaze, allowing the robot to use gaze behavior to signal its intent to engage in conversation. In this way, research has advanced techniques for engaging people through a combination of human-like modalities (Vázquez et al., 2017).

Research on robots interacting with multiple people has been widely studied, exploring aspects such as gestures, gaze behaviors, and turn-taking management. Rifinski et al. (2021) examined how a robot’s responsive gestures impact human-human interaction in multi-party settings. Their findings indicate that gaze and leaning gestures enhance interpersonal evaluation, leading to improved perceptions of conversation partners. Similarly, Shintani et al. (2024) analyzed the impact of a robot’s gaze control on the dynamics of multi-party conversations and personality expression. Their study experimentally validated how a humanoid robot can reproduce human-like gaze behavior by considering three key factors: conversational roles, turn-taking, and gaze aversion. Regarding turn-taking, Żarkowski, (2019) investigated how the social robot EMYS facilitates conversational flow in group interactions. Their study demonstrated that a robot’s effective management of speaking turns significantly enhances dialogue fluency. These studies highlight the growing interest in multi-party human-robot interaction and provide insights into key design considerations for robots engaging with multiple individuals in various social and conversational contexts.

These studies primarily focus on methods for humanoid robots to interact with people using various modalities, which may not be directly applicable to robots with non-human-like bodies. For non-humanoid robots, many interaction methods have been studied (Cha et al., 2018) using various means, such as gesture (Press and Erel, 2022), light (Cha et al., 2017), and augmented reality (Walker et al., 2018). To indicate to the surrounding partners whom the robot is addressing, existing methods have mainly relied on the robot’s body orientation and gaze direction, often displayed on a screen. Althaus et al. (2004) proposed a method in which a robot orients itself toward the center of a group when moving with multiple people. Kuzuoka et al. (2010) found that appropriate control of the robot’s torso and body orientation can achieve a positional relationship conducive to human conversation. Karreman et al. (2015) investigated the impact of body orientation when guiding partners through an exhibit. More recently, Takagi et al. (2023) examined the effects of the robot’s body orientation in multi-person conversations.

However, these methods have struggled to convey a clear sense that the robot is directly addressing specific partners nearby. In human-robot interaction, establishing “which person the robot is directly addressing” is essential for effective communication, as it forms the common ground necessary for interaction (Clark and Brennan, 1991). Unlike humans, robots face challenges in flexibly constructing such common ground. To address this issue, this study proposes a method where the robot uses a mounted projector to clearly indicate the intended addressee. This approach leverages the concept of physical co-presence, as discussed by Clark, to establish common ground through spatial referencing. In human-human interactions, gestures and eye gaze are commonly used to make spatial references to dialogue partners and objects, facilitating mutual understanding (Bangerter, 2004). Similarly, our method employs “projection on the ground” to identify the person being addressed, serving as a form of spatial referencing. This enables the robot to explicitly share the spatial reference of the intended addressee, especially in scenarios where traditional gestures are not feasible for the robot.

### 2.2 Research on projection robots

Recent advancements in the miniaturization and brightness of projectors have led to research on their use as interfaces for robots (Suzuki et al., 2022), with projection-based methods being classified as a type of Augmented Reality (AR)-based approach. To date, fundamental functionalities have been proposed for mounting projectors on robots to provide easily viewable projections from robots. J.-H. Lee (2007) proposed a method for presenting information to partners in the environment without location constraints by mounting a projector on a pan-tilt actuator on a robot. Donner et al. (2013) utilized a projector-equipped robot to guide partners, incorporating image distortion correction and self-localization capabilities. Additionally, various interfaces combining robots and projectors have been explored. Machino et al. (2006) proposed an efficient method for facilitating cooperative work by remote partners through projections from a robot. Saegusa (2017) developed a gait rehabilitation system using a mobile robot that projects the optimal positions for foot placement. Tamai et al. (2021) proposed a method that integrates movement and projection, using projection to guide a person’s standing position during the guidance process.

Several studies have also explored methods for robots to communicate their future behaviors to nearby partners using projectors, with the aim of achieving safe coexistence in daily environments. Matsumaru, (2008) proposed a method in which a robot uses a projector to display its future location by projecting information on movement speed and direction onto the floor. Coovert et al. (2014) examined the clarity and confidence level of pedestrians in understanding a robot’s intended direction when it projected arrows indicating its movement path onto the ground. Watanabe et al. (2015) introduced a wheelchair robot that projects its intended travel route, emphasizing the importance of an autonomous wheelchair sharing route information with both surrounding pedestrians and passengers.

However, these studies have only proposed methods for using projection to convey information from a robot to people, without addressing how to clarify the specific partner to whom the robot is speaking. In contrast, this study proposes a method in which the robot uses projection to envelop the intended conversation partner in light, clarifying whom it is addressing. This approach demonstrates an interface that utilizes projection to signal the start of interaction.

## 3 Using projection to clarify addressed partners

In this section, we propose a method to clearly indicate the partners to whom the robot is speaking by using a projector mounted on the robot. In the proposed method, the projection envelops both the robot and the intended conversation partner’s feet, clarifying the interaction partner and simultaneously enhancing the sense of participation in the interaction with the robot.

This study is conducted under the assumption that the robot can recognize the positions and postures of surrounding individuals, identify the people it intends to address, and approach them. This study specifically focuses on the phase in which the robot communicates with these partners, aiming to clearly convey to the surrounding people whom the robot is addressing.

### 3.1 Problems with robots starting to talk to partners

**FIGURE 1:**
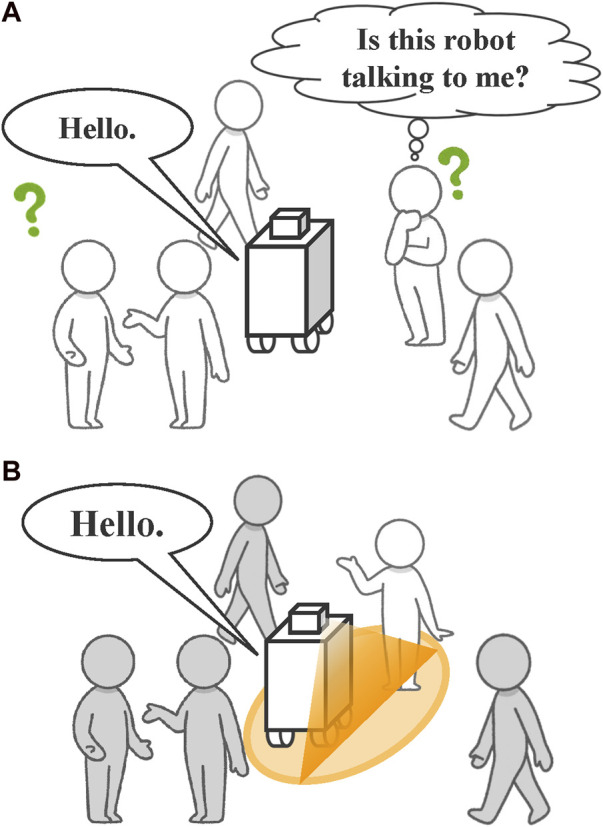
Difficulty faced by non-humanoid robots in clearly addressing individuals in the presence of others. **(A)** When the robot addresses a person, surrounding individuals cannot clearly identify whom the robot is speaking to, leading to confusion. **(B)** In the proposed method, the robot uses projection to clearly identify the individual it is addressing.

Figure 1A illustrates a non-humanoid robot attempting to speak to the partner on the right side of the figure. In this scenario, neither the intended conversation partner nor the other nearby partners can clearly understand whom the robot is addressing, resulting in unsuccessful dialogue initiation. Moreover, when addressing multiple people in such a situation, it is often unclear who is actively participating in the conversation. Therefore, a robot must be able to clarify its intended conversation partner and identify the partners engaged in the interaction.

Methods utilizing the robot’s body orientation and gaze direction on a display have been proposed to clarify the partners to whom a non-humanoid robot is speaking (Karreman et al., 2013). However, it remains challenging to clearly indicate the specific people the robot is addressing among surrounding partners. This study aims to address this issue by employing a projector, enabling the robot to clearly identify its intended conversation partners and share information effectively with nearby people (Figure 1B).

### 3.2 Using projection to clarify who a robot talks to

We propose a method in which a robot clearly indicates the area encompassing the partners it is interacting with by projecting an image onto the ground. Compared to the display method commonly used by robots coexisting with humans, ground projection offers the advantage of being easily visible from a wide range of directions, allowing the robot to clearly indicate multiple target partners simultaneously. Additionally, it is intuitively easy to understand, as it illuminates the area directly beneath each person’s feet.

**FIGURE 2:**
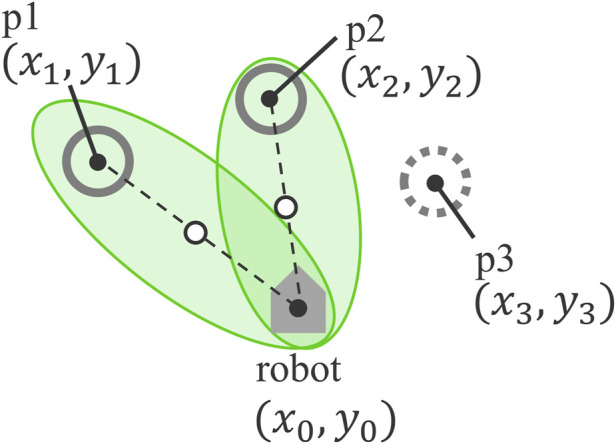
Positional relationship between the robot, the people being addressed, and the projected ellipse.

For each individual the robot addresses, it calculates an ellipse centered at the midpoint between the positions of the robot and the individual, projecting this ellipse onto the ground. Figure 2 illustrates an example of the positional relationship between the projected image and the people surrounding the robot. In the figure, p1 and p2 are the two individuals on the left, whom the robot is attempting to address, while p3 is not a target of the conversation. The white circle on the line connecting the center of gravity of the robot and individuals p1 and p2 in Figure 2 represents the midpoint between them. The ellipse encompassing the robot and the individual is centered on this midpoint, with the robot and the individual positioned at the foci of the ellipse. However, as the ellipse is projected within the range of the projector mounted on the robot, parts of the ellipse, such as the area behind the robot, may be truncated depending on the projector’s capabilities. This projection encompasses only the intended conversation partner(s), inviting them to participate and reinforcing the sensation that the robot is directly addressing them.

## 4 Experiment 1

To confirm the effectiveness of the proposed method in situations where a robot interacts with people, we tested it on a task in which a robot sequentially addressed multiple partners, asking each to move in turn.

### 4.1 Task

**FIGURE 3:**
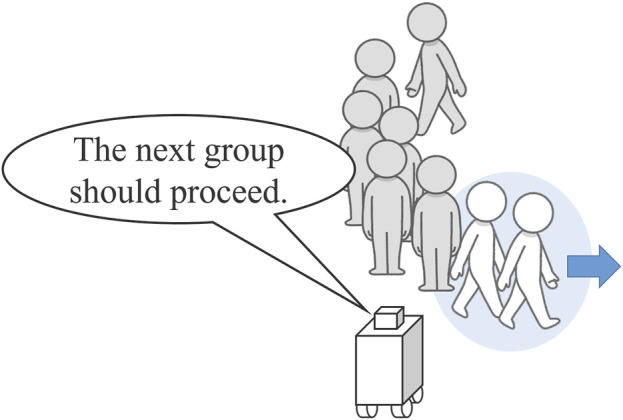
Guidance task by the robot, showing the robot instructing the first two individuals to proceed with entry.

In Experiment 1, we simulated a scenario in which the robot acted as a guide to manage facility entry, such as by restricting access and guiding partners in an orderly manner. The guiding robot’s task was to request that people advance in a single line as they were permitted entry into the facility (Figure 3). We evaluated the clarity and comfort of the instructions provided by the robot during this guidance.

**FIGURE 4:**
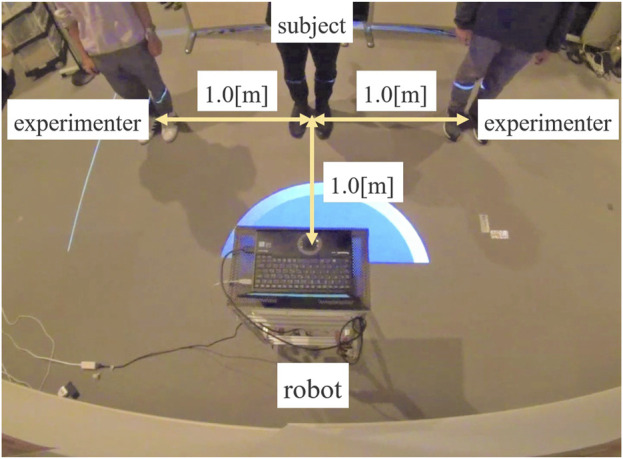
Experimental setup (Experiment 1).

Figure 4 illustrates the experimental setup. The robot stood in front of three partners aligned in a row and used voice commands to instruct one or two partners on the right side of the figure to move to the right. The partner in the center was the primary subject, while the two partners standing 1 m away from the subject were experimenters who consistently followed the robot’s instructions. When the robot addressed one or two partners on the right with the command, “Please move to the left,” it was necessary for the center participant to understand accurately whether they were included in the group instructed to move.

### 4.2 Experimental setup

#### 4.2.1 Robot

**FIGURE 5:**
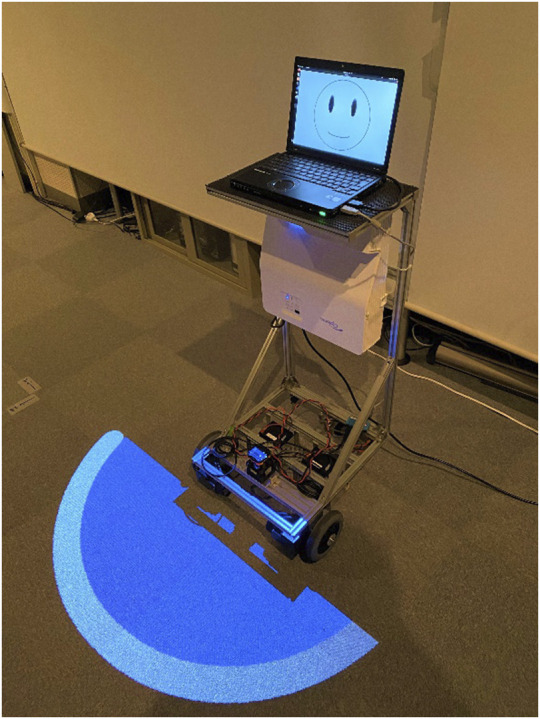
Projection robot used in the experiments.

**FIGURE 6:**
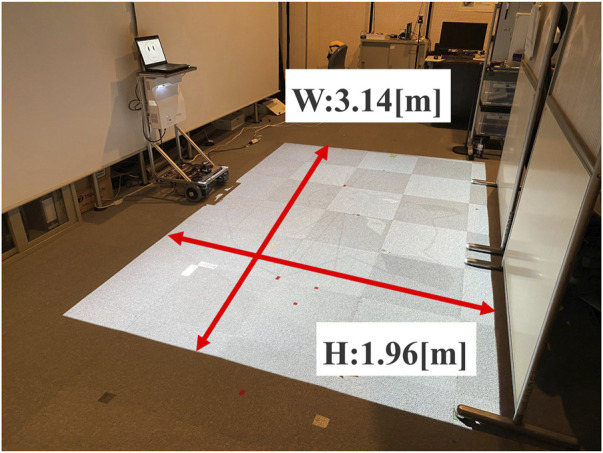
The extent of the projection range from the robot.

Figure 5 shows the projection robot used in the experiment. A projector (Optoma W340UST) was mounted on a mobile cart (T-frog Project i-Cart mini) for projection. The robot rotates at that location and turns its body to face the person to whom it is talking. To clarify the robot’s frontal orientation, an illustration of the robot’s face was displayed on the PC display on the robot. Figure 6 shows the projection range of the projector on the robot.

#### 4.2.2 People tracking system

We installed three LiDAR sensors (Hokuyo UTM-30LX) around the experimental environment to measure the positions of individuals within the area. To reliably measure the center of gravity of each person, the sensors were positioned at a height of 120 cm. The measurement process consisted of two steps: people detection and tracking. In the detection step, candidate individuals were identified through background subtraction and clustering. The system then detected an entity matching a typical person’s size and calculated its center of gravity. In the tracking step, a particle filter was applied to estimate each individual’s trajectory, producing a smoothed position at a rate of ten updates per second.

#### 4.2.3 Robot behavior

The robot detects a person’s location using the people tracking system and automatically executes a predetermined action to present the addressee via projection and body orientation. When the robot produces predefined speech utterances, the timing is manually triggered by the experimenter pressing a button.

### 4.3 System configuration

**FIGURE 7:**
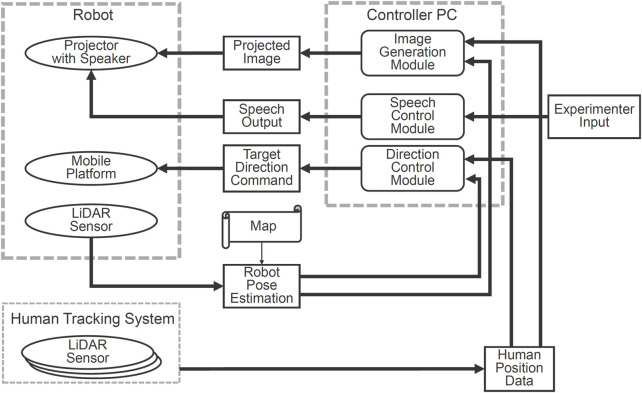
System configuration of the robot system.

Figure 7 illustrates the system configuration. The mobile cart estimates its own position and orientation using on-board range sensors and environmental map data, enabling it to move to a specified location and orientation. The robot control PC receives the positions of surrounding individuals from the human behavior measurement system, sends control commands to the cart to orient the robot’s body toward the designated individual, and generates a projection image on the floor, which is sent to the robot’s projector for display. For speech control, in this experiment, the experimenter used a remote control to trigger default speech, which was played through a speaker on the projector.

### 4.4 Conditions

A within-subject experimental design was used to investigate the effect of modality on the clarity with which the robot indicates the intended addressee (see Table 1). The independent variable was the modality used to indicate the intended addressee (Projection vs. Orientation). Each participant experienced both conditions in a counterbalanced order. The dependent variables included participant movement, subjective ratings on a seven-point Likert scale, and scores from the User Experience Questionnaire (UEQ; Laugwitz et al., 2008).

**TABLE 1:** Experiment design summary (experiment 1).

Independent variables		
Factor	Level 1	Level 2
Modality	Projection and orientation (proposed)	Orientation
Dependent variables		
Type	Measures	
Objective	Position	
Subjective (7-point Likert scale)	Clarity of understating Willingness to be guided by the robot	
Subjective (UEQ)	Pragmatic quality (Attractiveness, Perspicuity, Efficiency, Dependability)	

Projection-based condition: In addition to using body orientation, the robot indicated the person being addressed through projection, as described in the method proposed in Section 3.

Orientation-based condition: The robot indicated the person being addressed using only body orientation, while the projection displayed a fixed circular pattern centered on the robot.

The robot was tasked with addressing partners in a scenario where three people stood in a line in front of it (Figure 3). Guidance was provided in two distinct scenarios for each condition:

Scenario 1: The robot first instructed the first two partners to move, then, after a short interval, instructed the remaining two to move.

Scenario 2: The robot first instructed the first two partners to move, and then, after a short interval, instructed the remaining partner to move.

**FIGURE 8:**
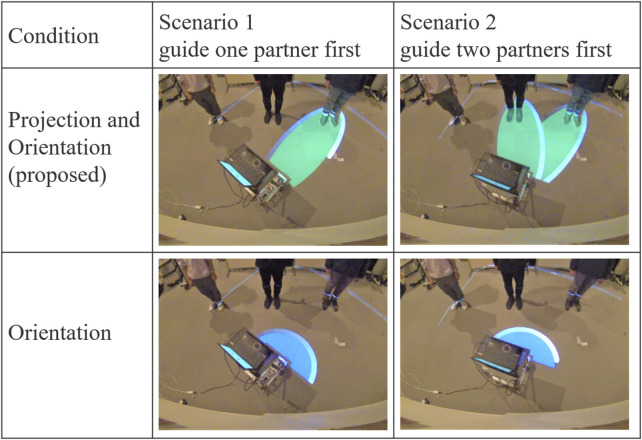
Conditions and scenarios in Experiment 1.

Figure 8 shows the behavior of the robot under each condition and scenario in Experiment 1. In this setup, the first and third partners in the line were experimenters who consistently followed the robot’s instructions—moving when instructed and remaining stationary otherwise. The primary subject stood as the second person in the lineup, and was required to move in response to the first instruction in Scenario 1 and to the second instruction in Scenario 2.

In both conditions, before speaking, the robot oriented itself toward the center of gravity of a single partner when addressing one person, and toward the midpoint between the positions of two partners when addressing two people.

### 4.5 Hypothesis

In situations where the robot is addressing specific partners around it, combining projection with body orientation is expected to enhance clarity in conveying who is being spoken to. Based on this, we formulated the following hypothesis:

Hypothesis 1 By clearly indicating the partner to whom the robot is speaking using the proposed method, recipients will understand the robot’s instructions more clearly and accurately than if only body orientation were used.

### 4.6 Measurements

Experimental participants completed a questionnaire to rate the clarity of understanding whom the robot was addressing and their willingness to be guided by the robot. Ratings were given on a seven-point scale, with seven indicating ‘very easy to understand’ and 1 indicating ‘very difficult to understand.’ The human behavior measurement system recorded the participants’ positions to verify whether they moved as instructed by the robot.

To evaluate the user experience of the robot service using projection, we also conducted a survey with the User Experience Questionnaire (UEQ) (Laugwitz et al., 2008). The questionnaire assessed six key aspects of user experience. In this experiment, participants responded to questions related to attractiveness, perspicuity, efficiency, and dependability aspects in the Japanese version of the UEQ.

Comparisons between conditions in the questionnaire-based evaluations were conducted using Wilcoxon’s signed-rank test. For the UEQ-based evaluations, comparisons between conditions were performed using Welch’s t-test, which accounts for unequal variances between two populations. In both tests, the significance level (α) was set to 0.05, which means that the results with p < 0.05 were considered statistically significant.

### 4.7 Participants

A total of 22 participants (1 woman and 21 men; average age: 23.1) took part in our experiment. The study was conducted from 19 December 2022, to 6 March 2023 at Hiroshima City University in a controlled laboratory environment. All participants were university students with a background in information science.

The study protocol was approved by the Ethics Committee of Hiroshima City University, Japan, and all participants provided written informed consent before participating in the study. They participated in both Experiment 1 and Experiment 2 sequentially, with each session lasting approximately 15 min and a 10-min break in between. Participants received monetary compensation for their participation.

### 4.8 Procedures

Participants were informed that the robot would use projections and body movements to deliver spoken instructions. Then they experienced how the robot behaves using both conditions in advance. Participants were told that the robot’s speech would include a greeting at the start of the session, followed by the instruction, ‘Please proceed to the left toward us.’ Additionally, participants were instructed to move to a designated position on the left near the robot when prompted and to remain there once they arrived. During each experiment, the participant stood at the center of a line of three people positioned in front of the robot.

The robot initiated the session with a voice greeting and guided participants through two scenarios in each condition. After completing the movements in each condition, participants filled out a questionnaire.

### 4.9 Results

**FIGURE 9:**
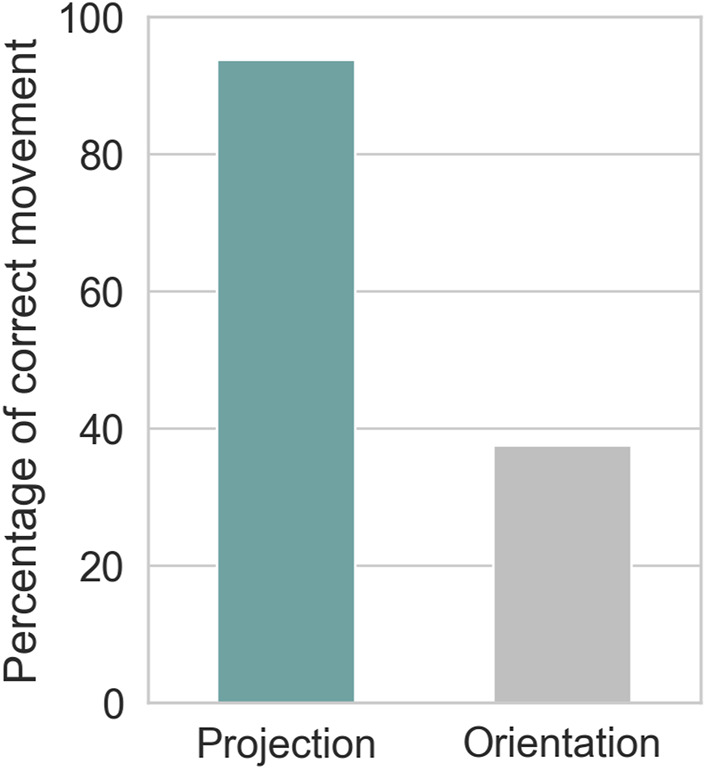
Proportion of participants who correctly followed the robot’s instructions.

Figure 9 shows the percentage of participants who correctly followed the robot’s instructions. In the projection-based condition, 93.8% of participants correctly interpreted and followed the robot’s instructions. In contrast, only 37.5% of participants correctly understood and responded to the instructions in the orientation-only condition, where the robot used only body orientation. In this latter condition, the robot failed to effectively convey its instructions, resulting in many participants acting contrary to the robot’s guidance.

**FIGURE 10:**
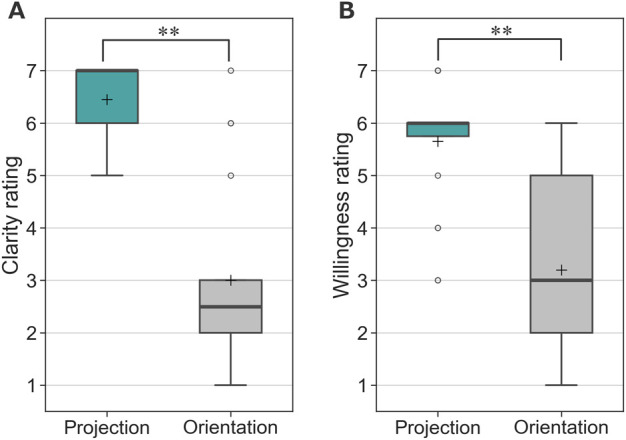
Questionnaire responses from participants in Experiment 1. **(A)** Clarity of the addressed person. **(B)** Willingness to be guided by the robot. (* indicates p < 0.05 and ** indicates p < 0.01).

Figure 10 shows the questionnaire evaluations. In terms of clarity regarding whom the robot was addressing, the proposed method scored significantly higher than the orientation-only condition, where the person was indicated solely by the robot’s orientation. A Wilcoxon signed-rank test confirmed a significant difference (V = 0, Z = 3.85, p < 0.05, p = 0.0001). The effect size, Cliff’s delta, was δ=−0.868 , indicating a large effect. These results suggest that the use of projection significantly improved the clarity of the robot’s addressee identification. Similarly, the projection-based condition also scored significantly higher than the orientation-only condition in terms of participants’ willingness to be guided by the robot. A Wilcoxon signed-rank test confirmed a significant difference (V = 0, Z = 3.73, p < 0.05, p = 0.0002). The effect size, Cliff’s delta, was δ=−0.770 , again indicating a large effect. These findings suggest a strong preference for the proposed method in guiding participants.

**FIGURE 11:**
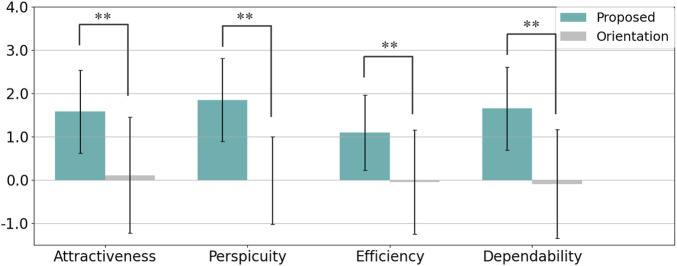
Summary of participants’ evaluations from the User Experience Questionnaire (UEQ) in Experiment 1 (* indicates p < 0.05 and ** indicates p < 0.01).

The User Experience Questionnaire (UEQ) was used to evaluate participants’ perceptions across four scales: Attractiveness, Perspicuity, Efficiency, and Dependability, with the results visualized in Figure 11. The reliability of each scale, measured by Cronbach’s alpha, is summarized in Table 2. A Welch’s t-test was conducted for each scale, with Cohen’s $d_{z}$ used to assess effect sizes. The results showed that the proposed method scored significantly higher than the orientation-only condition in all four scales: Attractiveness (p = 0.0003, $d_{z}=-1.29$ ), Perspicuity (p < 0. 0001, $d_{z}=-1.30$ ), Efficiency (p = 0.0014, $d_{z}=-1.04$ ), and Dependability (p = 0.0001, $d_{z}=-1.23$ ), all showing large effects. These findings indicate that the projection-based method strongly enhanced participants’ user experience, particularly in terms of clarity (Perspicuity) and overall appeal (Attractiveness), which exhibited the largest effects.

**TABLE 2:** Reliability analysis of UEQ scales: Cronbach’s alpha values (experiment 1).

UEQ scale	Projection	Orientation
Attractiveness	0.901	0.929
Perspicuity	0.808	0.598*
Efficiency	0.808	0.800
Dependability	0.698*	0.823

* Results of scales that need to be interpreted with caution.

Overall, in the projection-based condition, participants found the robot easier to understand, and more respondents expressed a preference to be guided by the robot.

## 5 Experiment 2

In Experiment 1, we evaluated whether the proposed method effectively indicates whether the robot is directly addressing a specific individual. In Experiment 2, we focused on a multi-person dialogue scenario, assessing the impressions of those who did not actively participate in the conversation. In situations where we are conversing with a robot, maintaining the sense that the robot is addressing you personally can enhance the feeling of inclusion in the conversation, which is essential for smooth communication. In this section, we examined the effect of projection in a scenario where two people ask a guide robot for directions, testing whether projection can effectively convey that the robot is addressing both partners. Additionally, we assessed the impact of projection on the impression of individuals who were present but not actively participating.

The same equipment used in Experiment 1 was employed to measure the behaviors of both the robot and the participants. Experiment 2 was conducted with the same participants from Experiment 1 and adhered to the same ethical procedures.

### 5.1 Task and environment

**FIGURE 12:**
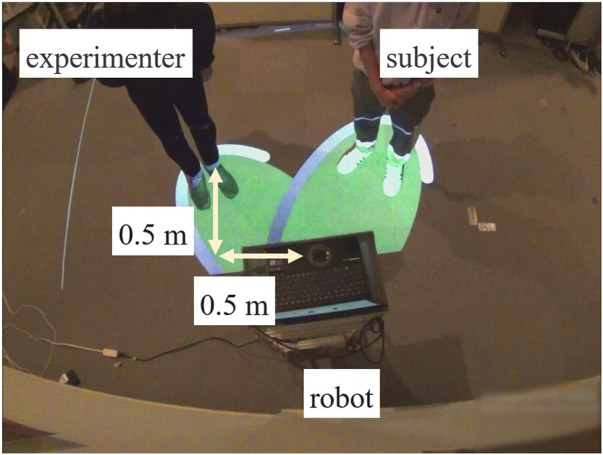
Experimental setup (Experiment 2).

The experiment simulated a scenario in which two people visit a commercial facility together, with one individual asking a guide robot for directions to their destination (Figure 12). In this setup, one of the two individuals was the participant, while the other was the experimenter. The experimenter directed the participant to approach the guide robot from the right side of the figure, then stopped at a predetermined position, greeted the robot, and asked for directions to the destination. The conversation between the experimenter (E) and the robot (R) followed a fixed set of predetermined dialogue, and an example of this dialogue is shown below. After the interaction, participants were asked to rate the extent to which they felt the robot was speaking to them.

E: Excuse me. Could you tell me the way to the student room?

R: The student room, correct? First, please exit this room, take the elevator, and go down to the 4th floor.

E: Where is the elevator?

R: The elevator is located to the left after you exit this room. After you get off the elevator, proceed down the connecting corridor, and you’ll find the student room to your right.

E: Thank you very much.

R: You’re welcome. Please feel free to ask if you need any further assistance.

### 5.2 Conditions

Experiment 2 employed a within-subject experimental design, identical to that of Experiment 1, to investigate the effect of modality on multi-party interaction (see Table 3). The independent variable was the modality used to indicate the intended addressee (Projection vs. Orientation), and each participant experienced both conditions in a counterbalanced order.

**TABLE 3:** Experiment design summary (experiment 2).

Independent variables		
Factor	Level 1	Level 2
Modality	Projection and orientation (proposed)	Orientation
Dependent variables		
Type	Measures	
Objective	Position	
Subjective (7-point Likert scale)	Feeling of participation Feeling of being noticed Clarity of understating Feeling ease in speaking	
Subjective (UEQ)	Pragmatic quality (Attractiveness, Perspicuity, Efficiency, Dependability) Hedonic quality (Stimulation, Novelty)	

As shown in Table 3, the dependent variables differed slightly from those in Experiment 1. While both experiments included participant movement and subjective ratings on a seven-point Likert scale, Experiment 2 specifically assessed participants’ sense of engagement in the conversation through additional questionnaire items. Additionally, Experiment 2 used the User Experience Questionnaire (UEQ) to evaluate all subscales, including Hedonic Quality, whereas Experiment 1 primarily focused on Pragmatic Quality.

In the scenario where two partners, the experimenter and the participant, approached the projection-equipped robot and the participant began asking questions, the robot conducted the conversation under the same two conditions as in Experiment 1. In both conditions, the robot was oriented toward the midpoint between the two partners, as measured by the human behavior measurement system. When the participants moved, the robot adjusted its orientation to follow the midpoint of their new positions.

**FIGURE 13:**
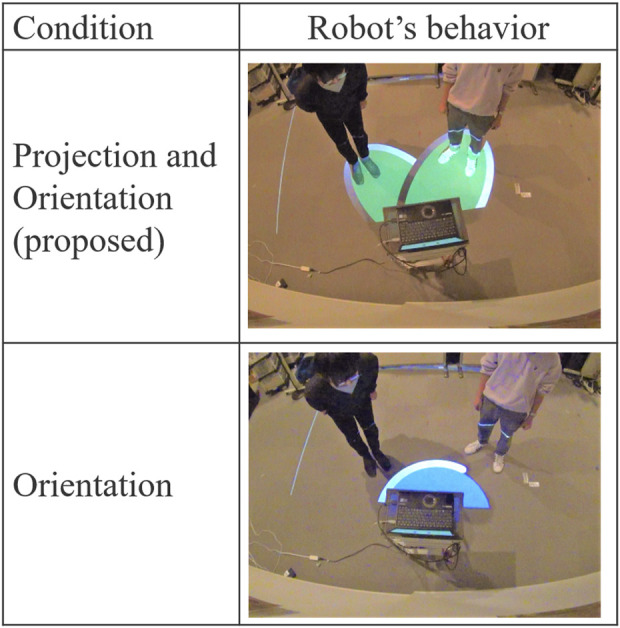
Conditions and the robot’s behavior in Experiment 2.

The robot began projecting once the participant started speaking. In Condition A (the proposed method), the projection was aligned with the measured positions of both partners and adjusted to follow any changes in their standing positions. In Condition B, the projection displayed a fixed pattern that did not adjust to the partners’ positions. Figure 13 shows the behavior of the robot under each condition and scenario in Experiment 2.

### 5.3 Hypothesis

In a situation where multiple people interact with a robot and only one person is conversing with the robot, it is expected that by using projection to indicate that the robot is addressing both partners, even the person who is not speaking will feel as though the robot is talking to them, creating a sense of participation in the conversation. We hypothesize that in Condition A, where the robot uses projection to address both people, compared to Condition B, the sense of participation will be enhanced for individuals who are present but not actively participating.

Hypothesis 2 In Condition A, where the robot uses projection to engage both partners, the sense of participation for individuals who are not actively participating in the conversation will be enhanced compared to Condition B, where the robot only faces both people.

Hypothesis 3 In Condition A, the sense that the robot is directly addressing the partner will be stronger compared to Condition B.

Hypothesis 4 In Condition A, partners will feel it is easier to talk to the robot compared to Condition B.

### 5.4 Measurements

After each conversation in both conditions, participants completed a questionnaire to rate their engagement in the dialogue, their perception of the robot’s awareness of them, their sense of being directly addressed by the robot, and their comfort level when interacting with the robot. As in Experiment 1, ratings were provided on a seven-point scale. An evaluation using the User Experience Questionnaire (UEQ) was also conducted. In this experiment, participants responded to all the questionnaire items, which assessed attractiveness, perspicuity, efficiency, dependability, stimulation, and novelty. Between-condition comparisons were conducted using the same statistical tests applied in Experiment 1. Comparisons between conditions in the questionnaire-based evaluations were conducted using Wilcoxon’s signed-rank test. For the UEQ-based evaluations, comparisons between conditions were performed using Welch’s t-test, which accounts for unequal variances between two populations. In both tests, the significance level (α) was set to 0.05, which means that the results with p < 0.05 were considered statistically significant. The human behavior measurement system recorded participants’ positions to verify their responses to the robot’s cues.

### 5.5 Participants

Participants from Experiment 1 also took part in Experiment 2. Thus, a total of 22 participants (1 woman and 21 men; average age: 23.1) participated in both experiments. The study protocol was approved by the Ethics Committee of Hiroshima City University, Japan, and all participants provided written informed consent before participation.

### 5.6 Procedures

Each experiment involved one participant and one experimenter. Participants were informed that the robot could provide directions verbally, engage in simple conversation, and occasionally use projection while speaking. They were also told that the experimenter and the participant knew each other and were together in a two-person situation heading toward a destination. Then they experienced how the robot behaves using both conditions in advance. From the initial position, the experimenter and the participant approached the robot, with the experimenter stopping at a predetermined position. The experimenter then engaged in a conversation with the robot, asking about the destination, as illustrated in Figure 13. Once the conversation concluded, the experimenter informed the participant that it marked the end of the interaction with the robot. The participant then completed a questionnaire.

### 5.7 Results

**FIGURE 14:**
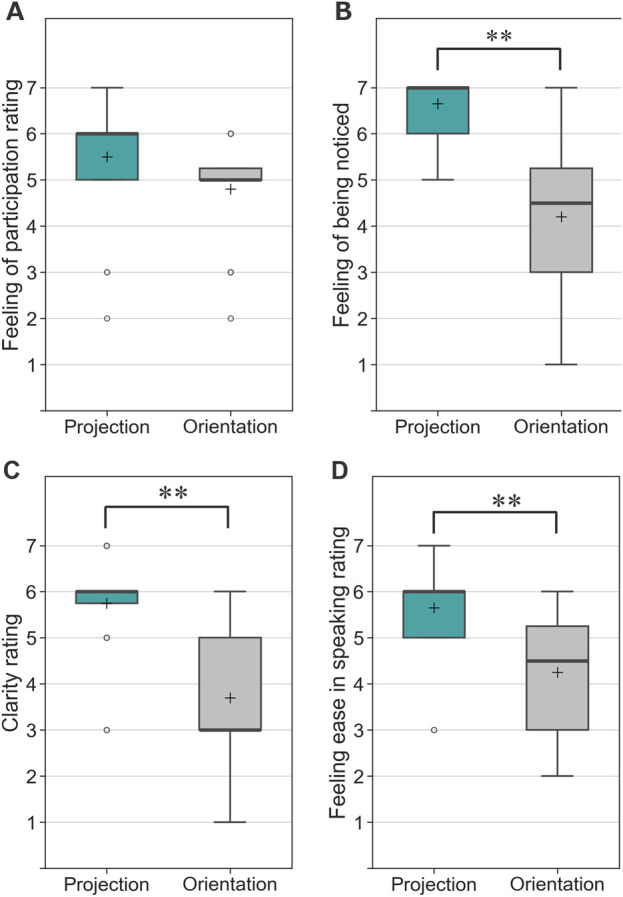
Questionnaire responses from participants in Experiment 2 (* indicates p < 0.05 and ** indicates p < 0.01). **(A)** Feeling of participation in the conversation. **(B)** Feeling of being noticed in the conversation. **(C)** Clarity of the addressed person. **(D)** Feeling of ease in speaking to the robot.

Figure 14 presents the impression ratings of the conversation with the robot. Both the median and mean values for the projection-based condition were higher than those for the orientation-only condition regarding the sense of participation in the dialogue (Q1). However, a Wilcoxon signed-rank test did not indicate statistical significance (V = 31.5, Z = 1.92, p = 0.055, Cliff′s delta=−0.433 ), though the effect size suggested a moderate effect.

In contrast, the projection-based condition significantly outperformed the orientation-only condition in the other three aspects: feeling of being noticed (Q2), clarity of understating (Q3), and feeling ease in speaking (Q4). A Wilcoxon signed-rank test confirmed significant differences for Q2 (V = 0, Z = 3.62, p < 0.05, p = 0.0003, Cliff’s delta = −0.853 ), Q3 (V = 0, Z = 3.51, p < 0.05, p = 0.0004, Cliff’s delta = −0.703 ), and Q4 (V = 4, Z = 3.34, p < 0.05, p = 0.0009, Cliff’s delta = −0.550 ), all indicating large effect sizes.

**FIGURE 15:**
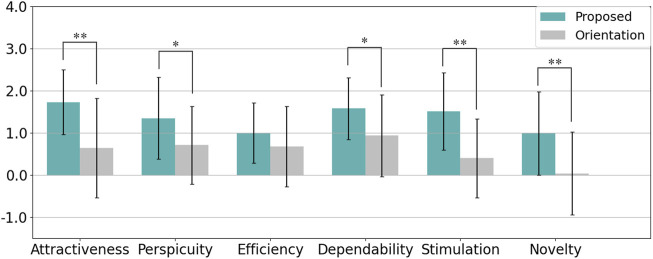
Summary of participants’ evaluations from the User Experience Questionnaire (UEQ) in Experiment 2 (* indicates p < 0.05 and ** indicates p < 0.01).

The User Experience Questionnaire (UEQ) was used to evaluate participants’ perceptions across six scales: Attractiveness, Perspicuity, Efficiency, Dependability, Stimulation, and Novelty, with the results visualized in Figure 15. The reliability of each scale, measured by Cronbach’s alpha, is summarized in Table 4. A Welch’s t-test was conducted for each scale, with Cohen’s $d_{z}$ used to assess effect sizes. The results showed that the proposed method scored significantly higher than the orientation-only condition in Attractiveness (p = 0.002, $d_{z}=-1.14$ ), Perspicuity (p = 0.039, $d_{z}=-0.63$ ), Dependability (p = 0.024, $d_{z}=-0.61$ ), Stimulation (p = 0.0005, $d_{z}=-1.08$ ), and Novelty (p = 0.004, $d_{z}=-0.93$ ), all showing moderate to large effects. Although Efficiency did not reach statistical significance (p = 0.230, $d_{z}=-0.38$ ), the effect size suggests a potential trend favoring the proposed method.

**TABLE 4:** Reliability analysis of UEQ scales: Cronbach’s alpha values (experiment 2).

UEQ scale	Projection	Orientation
Attractiveness	0.887	0.943
Perspicuity	0.844	0.673*
Efficiency	0.562*	0.783
Dependability	0.637*	0.793
Stimulation	0.915	0.788
Novelty	0.906	0.865

*Results of scales that need to be interpreted with caution.

## 6 Discussion

The primary contribution of this study is the proposal of a novel method for robots to clearly communicate their intended interaction partners through the use of projection. We introduced a projection-based approach that delineates the area around the robot and the partners it is addressing, enabling the robot to better convey its intention to interact. For successful human-robot interaction, it is crucial for robots to engage naturally with people and to sustain a feeling that the interaction is directed toward them. While significant research has focused on replicating human behaviors using humanoid robots, non-humanoid robots—such as delivery and security robots that are increasingly deployed in human environments—face unique challenges in initiating dialogue and signaling their intention to engage with people.

In Experiment 1, we examined whether projection could help a robot clearly indicate which partners in its vicinity it was addressing when making requests. Compared to using body orientation alone, the projection-based method significantly improved the clarity with which participants could identify whom the robot was addressing (p = 0.0001, δ=−0.868 ), effectively outlining the robot’s interaction range. Additionally, participants favored the projection-based guidance method (p = 0.0002, δ=−0.770 ), likely because the projection’s clarity in highlighting intended interaction partners enhanced their perception of the robot’s guidance.

Experiment 2 evaluated the proposed method in a scenario where multiple partners asked a guide robot for directions. In this experiment, we tested whether the projection-based approach could effectively enhance the sense of participation for individuals who were not actively participating. Results showed an increase in the sense of participation (p = 0.055, moderate effect), with significant improvements in the feeling that the robot was addressing both partners (p = 0.0004, δ=−0.703 ) and in the ease with which participants felt they could speak to the robot (p = 0.0009, δ=−0.550 ). This effect likely arises from the projection encompassing both partners alongside the robot, creating a shared perception of a three-way conversation.

The proposed method, which uses projection to clarify whom the robot is addressing and to enhance participants’ sense of involvement, holds promise as an approach for facilitating smooth interactions with non-humanoid robots. These robots often lack the capability to perform human-like gestures, such as hand or foot movements, facial expressions, or eye contact. While this study focused on tasks involving verbal communication, projection-based interfaces may prove beneficial for a range of other tasks as well. Many mobile robots assisting in daily life are equipped with displays; however, displays are challenging to view unless directly in front of them. In contrast, projector-based projections are visible from a wider range of angles, allowing shared access to the projected information. Leveraging this capacity for information delivery through projection may help robots perform tasks more effectively in everyday environments.

The robot used in this experiment displayed a simple face illustration on its screen to enhance the recognizability of its front, rather than to convey facial expressions or gaze direction. Therefore, while it remains uncertain whether our findings are applicable to robots without facial displays, they are likely transferable to robots with a clearly defined frontal orientation. Vázquez et al. (2017) investigated the effects of body orientation and gaze in group conversations using a robot capable of expressing facial expressions and gaze through back projection. In contrast, our robot’s face illustration served only to indicate body orientation. Thus, our findings are likely relevant to robots that do not rely on eye gaze information in the same way humans do.

In the experiments conducted in this study, the robot’s behavior was explained to the participants before the experiment began, and they had the opportunity to observe and interact with the robot. As a result, we have not examined how individuals unfamiliar with the robot would evaluate its behavior. Investigating how first-time users perceive and evaluate the robot remains an important direction for future research.

The visibility of projection-based interfaces varies depending on lighting conditions. While the experiments in this study were conducted indoors, where the projection was clearly visible, outdoor visibility may be limited with current equipment. The effectiveness of projection in bright environments, such as outdoor settings, depends on the capabilities of the projector. Future advancements in projection technology, such as laser projectors, may enhance visibility and enable more effective use in bright environments.

Further research challenges include sharing projected information with surrounding partners when obstacles are present between the robot and the person being addressed, or when the surrounding area is densely populated. In such crowded environments, it may be necessary to effectively combine projection with other modalities, such as robot motion control and auditory cues, to improve the effectiveness of the robot’s communication. Additionally, this study assumes that the robot can recognize and approach its intended interaction partners. Future work will consider integrating our approach with other methods currently under investigation for enabling robots to approach partners they intend to address. Verifying these comprehensive tasks remains a subject for future research.

This study has several limitations. One limitation is that the participant sample in this study was skewed toward male students with information science backgrounds, which may limit the generalizability of our findings. Prior research suggests that familiarity with technology and gender differences can influence perceptions of robots, potentially affecting user expectations and interaction preferences. Consequently, the impressions and evaluations in this study may not fully represent a more diverse population. Future studies should aim for a more balanced sample in terms of gender and academic background to enhance the breadth of user perspectives.

Another limitation of this study is that the interaction between the robot and humans is not entirely natural. In Experiment 1, we evaluated the robot’s interface in a scenario where it guided individuals in a queue into a store. However, since the study was conducted in a laboratory rather than an actual store, certain artificial constraints were introduced, such as requiring participants to wait at a predetermined location. These constraints were necessary to control experimental conditions but may limit generalizability to real-world environments. In Experiment 2, a scripted conversation between the experimenter and the robot was used, as the robot lacks the ability to respond dynamically to human utterances. Participants, who were not actively engaged in the conversation, were unaware that the dialogue was scripted during the interaction. After the conversation, they evaluated their own sense of participation and the extent to which they felt the robot was addressing them directly. While this controlled setting allowed us to assess the impact of the proposed method, it has not yet been tested in more flexible, natural conversations.

## 7 Conclusion

We proposed a projection-based method to enable robots to clearly communicate with their intended conversation partners. This method allows the robot to indicate who is participating in the dialogue by projecting an image on the ground that encompasses both the robot and the intended conversation partner. We evaluated this approach through two guidance tasks. Compared to the conventional method, where the robot merely orients its body toward the interlocutor, the projection-based method did not significantly enhance the sense of dialogue participation for non-speaking participants. However, it did lead to a significant improvement in the sense that the robot was addressing them directly and increased the ease with which they felt they could engage with the robot. We believe that robots employing various modalities, such as projection, movement, body direction, and auditory cues, to effectively convey their awareness and intentions can enhance human-robot interaction, particularly in everyday environments where robots coexist with humans. Research on effective information presentation methods by robots in daily life contexts remains an essential area of study.

## Data Availability

The original contributions presented in the study are included in the article/supplementary material, further inquiries can be directed to the corresponding author.
